# Persistence of Anti-S Titre among Healthcare Workers Vaccinated with BNT162b2 mRNA COVID-19

**DOI:** 10.3390/vaccines9090947

**Published:** 2021-08-25

**Authors:** Luca Coppeta, Giuseppina Somma, Cristiana Ferrari, Andrea Mazza, Stefano Rizza, Marco Trabucco Aurilio, Stefano Perrone, Andrea Magrini, Antonio Pietroiusti

**Affiliations:** 1 Department of Occupational Medicine, University of Rome Tor Vergata, Viale Oxford 81, 00133 Roma, Italy; luca.coppeta@ptvonline.it (L.C.); giuseppina.somma@ptvonline.it (G.S.); andrea.mazza@ptvonline.it (A.M.); stefano.perrone@ptvonline.it (S.P.); andrea.magrini@uniroma2.it (A.M.); pietroiu@uniroma2.it (A.P.); 2 Department of Traslational Medicine, University of Rome Tor Vergata, Viale Oxford 81, 00133 Roma, Italy; stefano.rizza@ptvonline.it; 3 Department of Medicine and Health Sciences “V. Tiberio”, University of Molise, 86100 Campobasso, Italy; marco.trabuccoaurilio@unimol.it

**Keywords:** SARS-CoV-2, healthcare workers, COVID-19, vaccine, mRNA vaccine, anti-S-RBD antibodies

## Abstract

The COVID-19 pandemic has led to health, social and economic consequences for public health systems. As a result, the development of safe and effective vaccines, in order to contain the infection quickly became a priority. The first vaccine approved by the Italian Agency for Drugs Authorization (AIFA) was the BNT162b2 mRNA vaccine, developed by BioNTech and Pfizer (Comirnaty). Comirnaty contains a molecule called messenger RNA (mRNA), which is a nucleoside-modified RNA that encodes the SARS-CoV-2 spike glycoprotein. Even if data from phase I suggest that vaccine induced antibodies can persist for up to six months following the second shot of BNT vaccine, data regarding the real duration of immunological protection are lacking. In this study, we aimed to evaluate the duration of serological protection by detecting the presence of anti-S-RBD (receptor-binding domain) antibodies for SARS-CoV-2 among a large group of healthcare workers (HCWs) three months after vaccination. 99% of HCWs had a detectable titre of anti-S SARS-CoV-2 antibodies 90 days after the second vaccine shot. Elderly operators showed significantly lower levels of protective antibodies when compared to the younger ones, thus they could become unprotected earlier than other operators.

## 1. Introduction

Healthcare workers (HCWs) are reported to be at higher risk of COVID-19 infection than the general population. Published papers have evaluated the role of individual and work characteristics associated with higher rates of infection among those subjects [1,2,3].

The COVID-19 pandemic has led to health, social and economic consequences for public health systems, thus the development of safe and effective vaccines, in order to contain the infection, has been fast-tracked worldwide [4].

Since 21 December 2020, four COVID-19 vaccines have been authorized by the European Medicine Agency (EMA) for emergency use: Comirnaty, Spikevax (previously COVID-19 vaccine Moderna), Vaxzevria (previously COVID-19 vaccine AstraZeneca) and COVID-19 vaccine Janssen [5]. In Italy, the first vaccine approved by the Italian Agency for Drugs Authorization (AIFA) was the BNT162b2 mRNA vaccine, developed by BioNTech and Pfizer (Comirnaty) [6]. The BNT162b2 mRNA vaccine is a nucleoside-modified RNA that encodes the severe acute respiratory syndrome coronavirus 2 (SARS-CoV-2)spike glycoprotein [7]. The RBD domain is the main target of neutralizing antibodies against coronaviruses of the spike protein. This protein (S protein) is a large transmembrane protein which helps the virus gain access to the cells. The S protein is composed of two subunits (S1 and S2). The receptor-binding domain (RBD) that is contained in domain S1 is the region that binds to receptor ACE2 allocated in the target cell membrane. The S protein of SARS-CoV-2 binds the ACE2 receptor with more affinity than that of SARS-COV [8]. Following natural responses to COVID-19 infection, antibodies are also generated against the nucleoprotein (N) of SARS-CoV-2 [9,10], but these antibodies are unable to neutralize the virus in humans.

It is administered intramuscularly in the deltoids in two 30-μg doses, with a recommended dose interval of 21 days, full protection against COVID-19 may not be achieved until 7 days after administration of the second dose. As is the case with other vaccines, BNT162b2 may not protect every recipient. BNT162b2 is comprised of nucleoside-modified mRNA formulated in lipid nanoparticles. The mRNA encodes the membrane-anchored, full-length SARS-CoV-2 spike protein and contains mutations, which stabilize the spike protein in an antigenically preferred, prefusion conformation. The lipid nanoparticles protect the non-replicating RNA from degradation and allow it to be delivered into host cells after intramuscular injection. Once inside host cells, the mRNA is translated into SARS-CoV-2 spike protein, which is expressed on the surface of the host cells. The transient expression of this spike antigen induces neutralizing antibody and cellular immune responses against it, which may confer protection against COVID-19 [11]. Data from clinical trials demonstrate that the vaccine is safe and effective [7,12]. Nevertheless, the rate of vaccination refusal among HCWs remains relevant for different reasons. As an example, we report the lack of confidence in vaccines (and fear of the potential hazards, including misconceptions about the risk of infection following vaccination), a poor understanding of the need to vaccinate (e.g., underestimation of disease severity), the fear of the potential side effects, political or religious influences, difficulties in accessing the vaccine and misinformation [13].

It has been found that even a single dose of BNT162b2 mRNA vaccine can give some level of protection against SARS-CoV-2 infection [14,15], even if further studies regarding the immunogenicity and efficacy of a single dose are still needed to confirm this result.

The duration of antibody responses againstSARS-CoV-2, both in convalescent and in vaccinated subjects, is one of the main research topics, since the duration of protection can be at least partially due to humoral immunity.

Data from convalescent patients showed that neutralizing antibody levels decline over time, between one to four months after symptom onset [16]. Moreover, the duration of the humoral immune response to SARS-CoV-2 among non-vaccinated convalescent patients, analyzed for twelve months, showed that the anti-spike IgG decline was positively associated with peak antibody titre and age, and inversely associated with WHO severity score [17].

Even if data from phase I studies suggest that vaccine induced antibodies can persist up to six months after a complete two doses cycle of vaccination, extensive data on the actual duration of immunological protection among vaccinated subjects are lacking. This question could be a crucial issue in the evaluation of effectiveness of vaccination among professionally exposed operators.

In this study, we aimed to evaluate the duration of serological protection by detecting the presence of anti-S-RBD (receptor-binding domain) antibodies for SARS-CoV-2 among a large group of HCWs three months after vaccination.

## 2. Materials and Methods

The study is a cross sectional one on routinely collected data, approved by the Ethics Committee of Policlinic Rome “Tor Vergata” (172/2021). Healthcare workers employed at a university hospital in Rome were enrolled in the study, after providing informed consent. We included all HCWs actively employed in the hospital during the study period who completed the vaccination cycle by 15 March 2021, with two doses of 0.3 mL of BNT162b2 mRNA vaccine administered to the deltoid muscle. None of the operators included in the study reported a confirmed COVID-19 infection before the inclusion in the study. A blood sample of 10 mL was collected from each participant, after a venipuncture of one of the arm’s veins (the cephalic vein or the median cubital vein or the basilic vein).

We determined the serum level of anti-S-RBD antibodies for SARS-CoV-2 using the Roche kit “Elecsys^®^” an immunoassay for the in vitro quantitative determination of antibody, which includes a recombinant protein representing the RBD domain of the spike protein in a double-antigen sandwich assay format, which allows detection of high affinity antibodies against SARS-CoV-2. A result ≥0.8 U/mL is considered positive, according to the manufacturer’s instructions.

A total of 300 HCWs were included in the study and underwent the serological test: 125 medical doctors (41.7%), 126 nurses (42.0%) and 49 other healthcare professionals (16.3%). Personal data and the result of serological evaluation were collected for all HCWs enrolled in the study.

Statistical analysis was performed using IBM^®^ SPSS^®^ Statistics (version 26). The level of significance was set at a *p*-value < 0.05.

We calculated the mean antibody titre for S-RBD of SARS-CoV-2 in the group of HCWs who completed the vaccination cycle with two doses. In order to assess the effect of gender and age on the duration of the antibody titre, we compared the data obtained in relation to those variables by means of analysis of variance (Anova) test, a linear regression analysis, identifying sources of variation in continuous data.

## 3. Results

We found the presence of the anti-spike S-RBD antibodies for SARS-CoV-2 in 298/300 (99.3%) subjects who underwent the serological screening, powered by the Occupational Medicine Department, more than three months after the second dose of vaccine.

Subjects who did not develop anti-spike S-RBD antibodies reported, when clinical history was being collected, to be affected by clinical conditions that may have weakened the immune response.

Descriptive characteristics of the study population by vaccination and serological status are reported in Table 1.

The mean time elapsed from the second shot of vaccine was 100 days (range 90–134 days).

**Figure 1:**
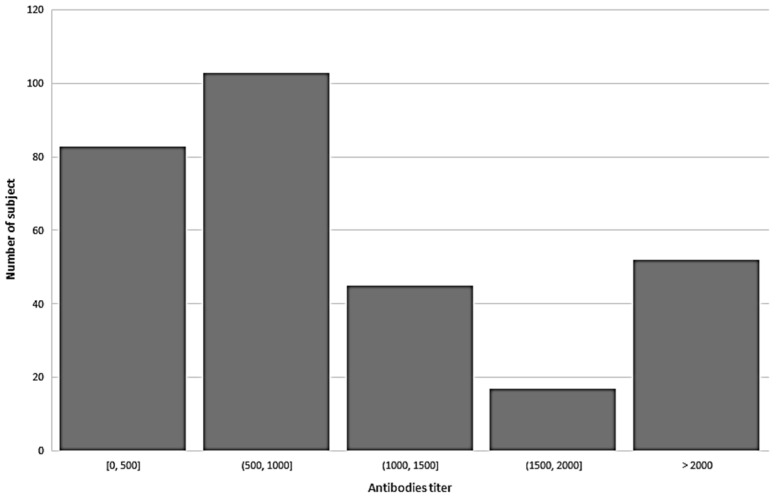
Distribution of antibody titre among study population.

We found that the mean anti-spike (S-RBD) antibodies for SARS-CoV-2 was 1032.3 ± 775.9 U/mL (range 0–2500 U/mL). Mean concentration was 1097 for females and 929.33 for male health operators (*p* < 0.05 at Anova test). See Figure 1 for the distribution of antibody titre among study population.

After performing a linear regression analysis, we found that the anti-spike (S-RBD) antibody titre was statistically related to the age of vaccinated operators (see Table 2). The average value of the titre among HCWs older than 50 years was 794.00 U/mL vs. 1130.80 found in the younger subjects.

## 4. Discussion

Detectable levels of IgM and IgG antibodies to SARS-CoV-2 can be found within one to two weeks following the onset of symptoms in most infected individuals [18].

Previous studies have reported that BNT162b2 elicits strong antibody response 7 days after the booster dose [19]; two doses of adenovirus 5 (Ad-5)-vectored vaccine were followed by a significant neutralizing antibody response to Sars-CoV2 at day 28 [20].

The persistence of the antibody responses to SARS-CoV-2 is currently a major study issue and relevant worldwide concern. Published studies on patients with previous SARS-CoV2 infection have shown that neutralizing antibody titre decreases between one year and two years after the viral infection [21].

A recent study analyzing various compartments of immune memory to SARS-CoV-2 in a high number of COVID-19 cases, found that serological response (IgG) against the S (spike) protein was stable for over six months after the infection and that Spike specific memory B cells were more abundant in this period than one month after the infection [22]. In a group of individuals who recovered from mild COVID-19 infection, it was found that neutralizing antibodies, IgG+ classical MBCs with BCRs that formed neutralizing antibodies, Th1 cytokine-producing CXCR5+ circulating Tfh and CXCR5− non-Tfh cells, proliferating CXCR3+ CD4+ memory cells, and IFN-γ-producing CD8+ T cells were present for at least three months [23].

Our data clearly show that a detectable level of neutralizing antibodies is present in almost all subjects enrolled in the study sample at about three months (90–134 days) after a complete vaccination with BNT162b2 (two doses of Comirnaty vaccine), and detectable level of anti-S-RBD antibodies for SARS-CoV-2 can be found up to four months after the second vaccination shot. Even if the level of neutralizing antibodies that is sufficient to confer protection against SARS-CoV-2 infection is unknown, we can suppose that subjects showing high antibody levels can be protected from SARS-CoV-2 infection.

Our findings confirm the results of previous studies: in a previous phase I trial on a small number of volunteers, it was found that serum-neutralizing antibodies were still detectable in all subjects of the study population at about 119 days after the second vaccine dose [24]. In a study on HCWs in Italy serum neutralizing activity remained detectable for a median time period of seven months following SARS-CoV-2 diagnosis in most of the study subjects [25].

Moreover, we found that the average antibody level was significantly related to the age of the healthcare workers, indeed the older subjects showed a significantly lower level of anti-S SARS-CoV-2 antibodies, compared to the younger ones.

In a recent study, older age was positively correlated with both higher IgG binding and neutralizing antibodies; the magnitude and duration of the antibody response after natural infection was lower and more variable in younger participants [26]. In a study on 255 HCWs, it was found that the increase of anti-Spike-RBD IgG following vaccination with BNT162b2 mRNA vaccine was lower in subjects aged 51–70 years compared to those aged 25–50 years [27]. In a recent paper on 33 healthy adults, antibodies elicited by the mRNA vaccine were detectable 6 months after the second dose and their title was lower among older subjects, although the difference was not statistically significant [28].

In our knowledge, this is the first “in vivo” observation regarding the immune response (anti-spike S-RBD antibodies) to COVID-19 vaccine in a relevant number of HCWs, in relation to age. Our findings raise questions on the waning immunity in elderly subjects that account for about one third of our working population. Moreover, elderly subjects showing lower antibody levels represent the population group at higher risk of severe clinical manifestation, hospitalization and complication in case of COVID-19 infection.

The main limitation of our study is the cross-sectional model, which did not allow us to evaluate the trend of antibody levels over time; this could be studied in a further analysis. Moreover, the spike specific memory B and T cells response was not evaluated, thus we cannot assess for an immune response other than the serological one. Another limit of our study is that the observation period was limited to about three months, further studies are needed to investigate the decrease in antibody levels after a longer period of vaccination. Furthermore, we did not evaluate the baseline antibody levels of the subjects enrolled in the study and therefore could not compare the effect of vaccine in different subgroups. We also did not evaluate the IgG titre at different times after vaccination, making it difficult to evaluate the trend in the antibody decrease, although was not the purpose of our study. Moreover, we did not systematically record data regarding the presence of subjects taking medications that could potentially affect the immune system. However, we can reasonably exclude the presence of pharmacologically immunosuppressed operators in the study population, since HCWs suffering from known immunosuppressive conditions were excluded from hospital work during the COVID-19 pandemic. Finally, as a matter of fact, we do not actually know the level of anti-S-RBD SARS-CoV-2 antibody titre that can be considered protective, so we cannot establish the effective serological immunity of our population. Therefore, future studies are needed to investigate the neutralizing potential of antibodies that would provide real protection from COVID-19 infection.

## 5. Conclusions

Our study showed that 99.3% of HCWs vaccinated with two doses of BNT162b2 vaccine had a detectable titre of anti-S-RBD SARS-CoV-2 antibodies 90 days after the second shot of vaccine. Elderly operators showed significantly lower levels of protective antibodies when compared to the younger ones. The occupational medical service should monitor antibody levels over time in order to assess the possible decrease in antibody level among these subjects since they could become unprotected earlier than the younger operators.

## Figures and Tables

**Table 1:** Study population characteristics by vaccination and serological status.

Age		**N (%)**	**Mean ± Standard Deviation**	**Range**	
			43.00 ± 11.0	21	75
Gender	Female	184 (61,33)			
	Male	116 (38,67)			
Serology for anti-S	Positive	298 (99,33)			
	Negative	2 (0,67)			
Anti-Spike RDB Titre			1032 ± 756	0	2500
Job task					
	Medical doctor	125 (41,67)			
	Nurses	126 (42,00)			
	Others HCWs *	49 (16,33)			
Days since last vaccine dose			101 ± 18	90	134

* Other HCWs: lab technicians, radiology technicians, perfusionist technicians, neurophysiopathology technicians, cardiocirculatory physiopathology technicians, psychologists, biologists, dieticians, dental hygienists, speech therapists, orthoptists, pharmacists.

**Table 2:** Factors influencing the anti-SARS-CoV-2 antibody titre (linear regression analysis).

Factors				95.0% Confidence Interval for B	
	beta	t	Sig.	Lower Bound	Upper Bound
**Age**	−21.191	−5.533	0.000	−28.728	−13.654
**Days since last vaccine dose**	0.497	0.217	0.828	−4.002	4.997
**Gender**	−147.951	−1.734	0.084	−315.852	19.950
